# Thiol Signalling Network with an Eye to Diabetes

**DOI:** 10.3390/molecules15128890

**Published:** 2010-12-06

**Authors:** Elena Matteucci, Ottavio Giampietro

**Affiliations:** Department of Internal Medicine, University of Pisa, Pisa, Italy; E-Mail: o.giampietro@med.unipi.it

**Keywords:** oxidation-reduction, sulphydryl compounds, *N*-acetylcysteine, diabetes mellitus, arterial hypertension

## Abstract

Redox regulatory system controls normal cellular functions. Controlled changes in redox couples potential serve as components for signal transduction, similarly to the phosphorylation cascade. Cellular redox biology requires both compartimentalisation and communication of redox systems: the thermodynamic disequilibrium of the major redox switches allows rapid and sensitive responses to perturbations in redox environments. The many oxidation states of sulphur are found in numerous sulphur species with distinct functional groups (thiols, disulphides, polysulphides, sulphenic, sulphinic and sulphonic acids, *etc.*), which participate in a complicated network of sulphur-based redox events. Human diseases such as diabetes mellitus and its cardiovascular complications have been associated with increased production of reactive oxygen species and perturbations of thiol redox homeostasis. The review surveys literature related to some etiopathogenic aspects and therapeutic perspectives. The dual toxic-protective property of sulphydryl-donor molecules in experimental settings proposes the general problem of designing antioxidants for therapeutic use.

## 1. Sulphur-Containing Amino Acids

In the body of the reference man sulphur (S) is present in about 140 g [1]. The two principal dietary sources of S-containing compounds in human nutrition are liquids (SO_4_^2−^ in drinking waters) and solid food products (organic S in the form of cysteine and methionine). The intake of methionine might be sufficient to meet the metabolic needs of an adult individual for exogenous sulphur, since cysteine is a metabolic product of methionine catabolism. However, given the uncertainties in literature, WHO/FAO/UNU Experts decided that there should be separate recommendations for methionine and cysteine intake (10.4 mg/kg per day and 4.1 mg/kg per day, respectively) [2]. Total body sulphur partitions into three body compartments: 1) a nonmetabolic, poorly exchangeable pool in keratin, collagen, connective tissue, cartilagen, tendons, *etc.*; 2) labile compounds and non-protein-bound thiols, such as glutathione (GSH); and 3) the muscle mass. 

**Figure 1:**
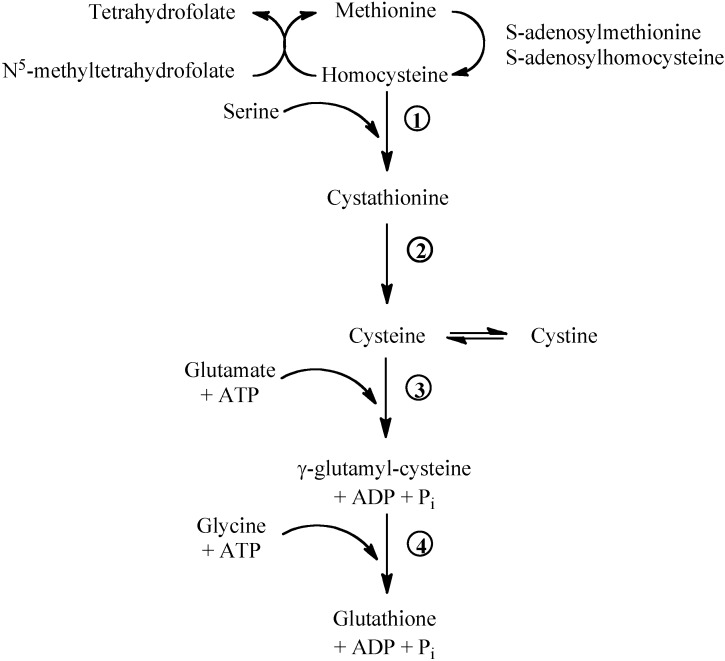
Methionine/glutathione transsulfuration pathway. Methionine is converted by methionine adenosyltransferase to *S*-adenosylmethionine, which is then converted to *S*-adenosylhomocysteine by methyltransferase. S-adenosylhomocysteine hydrolase catalyses the reversibile hydrolysis of S-adenosylhomocysteine to homocysteine and adenosine. Methionine can be regenerated from homocysteine via 5-methyltetrahydrofolate-homocysteine methyltransferase in the presence of N^5^-methyl-tetrahydrofolate and the cofactor cobalamine. The vitamin B6-dependent enzyme cystathionine-β-synthase (1) combines homocysteine and serine to produce cystathionine. Cystathionine is broken down to cysteine and α-ketobutyrat via the B6-dependent enzyme cystathionine-γ-lyase (2). Gamma-glutamylcysteine is synthesized from L-glutamate and cysteine via the enzyme γ-glutamylcysteine synthase (3). This reaction is the rate-limiting step in glutathione synthesis. Successively, glycine is added to the C-terminal of γ-glutamylcysteine via the enzyme glutathione synthase (4).

Methionine breakdown occurs by transmethylation to homocysteine that can be remethylated to methionine by the folate- and vitamin B_12_-dependent methionine synthase (Figure 1). Homocysteine can donate the sulphur group to serine and form cystathionine. Cystathionine can be broken down to cysteine and α-ketoglutarate. Cysteine, cystine and methionine are glycogenic amino acids: their carbon skeleton can be completely oxidised and the amino nitrogens incorporated into urea via the Krebs-Henseleit cycle.

So far we have no validated biomarker for S-containing amino acid deficiency [3]. Despite the many drawbacks, the plasma or intracellular concentration and redox state of GSH are presently considered surrogate endpoints of clinical studies. The ratio of GSH/GSSG (glutathione disulphide) allows estimating the redox state of a system. The determination of the molar concentrations of GSH and GSSG in homogeneous fluids, such as plasma, does assess the redox environment, while their compartmentation in cells or tissues may pose some problems. The measurement of total content of GSH and GSSG in many cell types reflects principally the redox environment of the cytosol (depending on the portion of cell volume occupied by the nucleus). However, the numerous proteins containing sulphydryl groups present as thiols (-SH), disulphides (PS-SH), or as mixed disulphides (PS-SG) can influence the redox environment of the cell [4].

## 2. Thiol/Disulphide Redox Systems

The many oxidation states of sulphur are found in numerous sulphur species with distinct S-containing functional groups (thiols, disulphides, polysulphides, sulphenic, sulphinic and sulphonic acids, *etc.*), which participate in a complicated network of sulphur-based redox events [5], whose compartmentation has been extensively reviewed [6]. Moreover, the metal-binding properties of sulphur compounds allow the generation of bioinorganic metal complexes and iron-sulphur (Fe-S) clusters are essential electron carriers and enzyme cofactors involved in the mitochondrial respiratory chain, mitochondrial iron homeostasis, tricarboxylic acid cycle enzymes, and DNA repair [7].

The sulphur in cysteine occurs in the oxidation state of -2. Consequently, cysteine in proteins plays not only structural roles, but can participate in redox reactions, such as thiol-disulphide exchange, single- or two-electron transfer, hydrogen-atom transfer, and nucleophilic substitution [5]. These redox reactions are favoured when protein folding provides appropriated environment for stabilization of cysteine sulphydryl group in the anionic form [8]. Indeed, the electrostatic milieu of cysteine thiols in proteins can have a substantial influence on the rates of thiol-disulphide exchange reactions [9]. Disulphides themselves, usually viewed as structurally stabilising elements in proteins, are recently emerging as members of the redox-sensitive thiol-based regulatory switch family that is involved in preservation, restoration, and modulation of protein function [10].

Thioredoxins, glutaredoxins, and protein disulphide isomerases are thiol/disulphide oxidoreductases that are structurally similar and have a conserved thioredoxin domain with the typical Cys-X-X-Cys active site motif [11]. However, the large thioredoxin-like superfamily includes both protein disulphide oxidoreductases containing a classic thioredoxin domain and members that do not function as oxidoreductases but contain a thioredoxin-fold domain (Table 1) [12].

**Table 1:** The clan Thioredoxin-like (CL0172) currently includes 27 member families related to the thioredoxin family (Pfam protein families database) [12].

FAMILY	PF	Annotation
AhpC-TSA	00578	Proteins related to alkyl hydroperoxide reductase and thiol specific antioxidant
ArsC	03960	Arsenate reductase-like
Calsequestrin	01216	Calcium-binding protein (sarcoplasmic reticulum)
DIM1	02966	Mitosis protein DIM1
DSBA	01323	Disulfide oxidoreductase
DUF1525	07511	Protein of unknown function
DUF1687	07955	Protein of unknown function
DUF255	03190	Protein of unknown function
DUF836	05768	Glutaredoxin-like domain
DUF899	05988	Bacterial protein of unknown function
DUF953	06110	Eukaryotic protein of unknown function
ERp29_N	07912	Endoplasmic reticulum protein ERp29, N-terminal domain
Glutaredoxin	00462	Glutaredoxins or thioltransferases
GSHPx	00255	Glutathione peroxidase
GST_N	02798	Glutathione S-transferase, N-terminal domain
HyaE	07449	Hydrogenase-1 expression protein
KaiB	07689	Cyanobacterial circadian clock protein
OST3/OST6	04756	Oligosaccharyltransferase subunits
Phosducin	02114	Phosphoprotein (complessed with transducin)
Redoxin	08534	Peroxiredoxin, thioredoxin and glutaredoxin proteins
SCO1-SenC	02630	Involved in biogenesis of respiratory and photosynthetic systems
SeIP_N	04592	Selenoprotein P, N terminal region
SH3BGR	04908	SH_3_-binding, glutamic acid-rich protein
T4_deiodinase	00837	Iodothyronine deiodinase
Thioredoxin	00085	Disulphide-containing redox proteins
Tom37	10568	Outer mitochondrial membrane transport complex protein
YtfJ_HI0045	09695	Bacterial protein of unknown function

*Thioredoxins* (whose active site contains typically the sequence Cys-Gly-Pro-Cys) participate in redox reactions by the reversible oxidation of an active centre disulphide bond. The thioredoxin 1 system is found in cytoplasm and nucleus, whereas thioredoxin 2 is specific to the mitochondria. Due to a very negative redox potential, thioredoxins reduce disulphides in proteins and in the process form an intramolecular disulphide at the active site. Reduced thioredoxin is regenerated by thioredoxin reductase in a reaction that oxidises NADPH [13]. Recently, a thioredoxin-interacting protein (TXNIP) has been identified that binds to and inhibits thioredoxin. TXNIP is a highly glucose inducible proapoptotic factor in β-cell and could be involved in the regulation of β-cell mass and diabetes [13]. Thioredoxins and related oxidoreductases may link to a protein and act as molecular chaperone until it reaches a stable folding state. They can also interact with molecular chaperones and peroxiredoxins. The chaperone activity may be redox regulated as well as independent of the active site cysteines [11]. 

Like thioredoxins, *glutaredoxins* catalyse the reduction of disulphide bonds in target proteins. Dithiol glutaredoxins (typical active site Cys-Pro-Tyr-Cys) are thiol-disulphide oxidoreductases kept reduced by glutathione. They reduce disulphide bridges by using two different mechanisms. The 2-Cys mechanism requires both cysteine residues of the active site, whereas the 1-Cys mechanism requires only the most N-terminal cysteine and serves to reduce mixed disulphides. Glutaredoxins might use a 1-Cys mechanism of action to catalyse the reduction of mixed disulphides with glutathione [11]. Some glutaredoxin have been implied in Fe-S cluster biosynthesis. Indeed, a splicing defect of glutaredoxin 5 has been identified as the cause of a sideroblastic-like microcytic anemia in humans [7]. Moreover, recent evidence suggests that high glucose induces glutaredoxin in rat retinal Müller cells, with concomitant NF-kB activation and increased expression of intercellular adhesion molecule-1. Thus, redox regulation by glutaredoxin in retinal glial cells is perturbed by diabetes leading to a pro-inflammatory response [15]. 

*Protein disulphide isomerases* (PDI) possess much lower negative redox potential then thioredoxin and glutaredoxin and, thus, preferentially catalyse the formation of disulphide linkages in proteins [8,16]. The more oxidising redox state of the endoplasmic reticulum (ER), where PDI are found, favours only the formation and isomerisation of disulphide bonds that occurs during the folding process and is essential for proteins to reach their native structure. The Ero1 enzymes are thought to reoxidise the PDI active site concomitantly with the conversion of molecular oxygen to hydrogen peroxide. Beyond protein maturation, additional functions of PDI include ER-associated degradation, hormone binding and reservoir, vitamin K1 regeneration, and dihydroascorbate reduction [16].

Major changes in the redox status of the ER and its redox chaperones are now being explored in diabetes, which could contribute to the defective protein secretion. In rat liver microsomes, after four weeks of streptozotocin-induced diabetes, both PDI-58 and ERp57 had a large fraction of the reduced enzyme form with a concomitant decrease of the oxidoreductase activity [17]. Changes in abundance of chaperone-like proteins have been observed in skin fibroblasts from long-term type 1 diabetes patients with nephropathy. Among molecules controlling protein folding, assembly and turnover, was changed the expression of PDI A3, A7, and A39 as well as heat shock protein 71 kD or HSP71, HSP60, and HSP27) [18].

*S-glutathionylation* of cysteine residues has been initially considered to be an antioxidant defence, able to protect sensitive cysteines from further oxidation. Successively, it has received attention as a posttranslational modification used as regulatory and signalling mechanism [19]. The susceptibility of a cysteine to glutathionylation is determined by its accessibility (due to the dimension of GSH molecule) and reactivity (influenced by the surrounding amino acids). Glutaredoxins containing one cysteine in the active site have been especially implied as dethiolases. The peroxidative cysteine at the N-terminal part of the protein is oxidised to sulphenic acid. The resolving cysteine, when present, reacts with the sulphenic acid generating a disulphide bridge.

Liquid chromatography/electrospray ionization-mass spectrometry demonstrated that glutathionylated haemoglobin levels were markedly increased in diabetic patients with microangiopathy [20]. Reduced inhibition of protein tyrosine phosphatase 1B via glutathionylation (due to high GSH peroxidase 1 activity) or long-term intake of Se supplements exceeding the requirements could promote the development of obesity and diabetes [21]. 

Unlike thiol/disulphide oxidoreductases, thiol-dependent, selenium-free and heme-free peroxidases reduce both hydroperoxides and peroxynitrite through a reactive cysteine. *Peroxiredoxins* may contain two conserved cysteines or only one conserved cysteine. Prx activity is regulated at the level of gene expression and by post-translational mechanisms such as phosphorylation, oligomerisation, non covalent binding of ligands, and sulphinylation of peroxidative cysteine. Cysteine sulphinylation has been shown to be reversible in eukaryotes by reductases, thus implying Prx in modulating hydrogen peroxide signalling [22]. As mentioned earlier, Prx can switch from a peroxidase activity to a chaperone function resulting from peroxidative cysteine overoxidation that prevents oxidant-induced damage. In transgenic mice, reduction of mitochondrial hydrogen peroxide by overexpressing Prx3 improves glucose tolerance probably due to glycogen synthase kinase 3 inhibition [23]. 

Finally, *S-nitrosation*, *i.e.* the oxidative addition of nitric oxide (NO) to cysteine residues of proteins, has been proposed as a cGMP-independent signalling pathway that mediates a number of actions of the NO group in various biological processes. Insulin resistance induced by increased inducible NO synthase in obesity may be related to *S*-nitrosation of insulin signalling proteins, insulin receptor, insulin receptor substrate 1 and protein kinase B (Akt) [24]. 

## 3. Glutathione and Plasma Thiols

Glutathione is the prevalent intracellular thiol [25]. Having its pKa 9.2, most of the cellular GSH is in the reduced form. The tripeptide (L-γ-glutamyl-L-cysteinylglycine) is synthesised in the cytoplasm by the consecutive actions of γ-glutamylcysteine synthase (the rate-limiting enzyme, inhibited by GSH) and GSH synthase (Figure 1) and is then exchanged with other intracellular compartments, mitochondria included. GSH breakdown is catalysed by γ-glutamyltranspeptidase, which transfers the γ-glutamyl moiety to acceptors and is localised on the external surface of cell membrane and in plasma. Intracellular GSH may be converted to GSSG by GSH peroxidase, which reduces H_2_O_2_ and other peroxides. GSSG is in turn reduced to GSH by the enzyme GSSG reductase in the presence of NADPH. Thus, intracellular GSH kinetics are affected by precursor availability, synthetic capacity, redox balance, and outward transport rate from cells. GSH is involved in vital processes: scavenging of highly oxidising species both directly and indirectly through enzymatic reactions, reaction with electrophiles, metabolites, and xenobiotics to form mercapturates, conjugation with NO to form an S-nitroso-glutathione adduct, removal of formaldehyde, conversion of prostaglandin H_2_ into prostaglandins D_2_ and E_2_, and glutathionylation of proteins. 

Plasma concentration of GSH depends on the balance between secretion from the liver and degradation in the kidney. Circulating GSH, GSSG, and CySSG (the disulphide of cysteine and GSH) are hydrolised in kidney to release cysteine and cystine. Diurnal variation of hepatic and plasma GSH concentrations in relation to sulphur amino acid dietary intake has been described in animals and humans. Plasma Cys/CySS (cystine) and GSH/GSSG redox mirrored the changes in GSH concentrations with mean differences between maximal and minimal redox state values of 6 and 4.5 mV, respectively. These differences could be functionally important in redox-dependent processes such as monocyte adhesion [26]. Indeed, in LDL receptor-deficient mice, the macrophage glutathione reduction potential was a strong predictor of macrophage chemotaxis. Thiol oxidative stress enhanced macrophage recruitment into vascular and renal lesions by increasing the responsiveness of macrophages to chemoattractants and macrophage chemotactic activity increased with increasing levels of metabolic stress [27].

Patients with type 1 diabetes mellitus have lower levels of both erythrocyte GSH and plasma sulphydryl groups. Reduced glutathione concentration in erythrocytes shows negative correlations with short- and mean-term metabolic control parameters, such as fasting plasma glucose and fructosamine, rather than with long-term HbA1c. Plasma thiols are decreased not only in patients with type 1 diabetes but also in their first-degree relatives and the oxidative stress observed in type 1 families is related to immunologic hallmarks (decreased peripheral numbers of monocytes as well as cells bearing a CD4^+^CD8^+^, CD23^+^CD25^+^, and CD25^+^ phenotype) [28,29].

Increased oxidative stress and associated oxidative damage are considered mediators of vascular injury in cardiovascular pathologies, including hypertension and atherosclerosis. Indeed, plasma SH-groups content has been found significantly reduced, and byproducts of oxidative protein damage increased, in patients with essential hypertension [30]. We have identified an abnormal blood pressure response to exercise testing in asymptomatic normotensive non-diabetic relatives of type 1 diabetics, that was associated with clinical characteristics of the metabolic syndrome (high BMI and LDL cholesterol), markers of oxidation (low plasma thiols), and familiarity for diabetic nephropathy [31]. Similarly, recent evidence has confirmed a significant correlation between oxidised thiol species, cystine and the mixed disulphide formed in the reaction of GSH with cystine, and endothelial function assessed by flow-mediated vasodilation [32].

Although most cellular GSH is in the cytoplasm, a distinctly regulated pool is present in mitochondria. Organic anion carriers, such as dicarboxylate carrier and oxoglutarate carrier, could function in the transport of the negatively charged GSH molecule across the mitochondrial inner membrane. Type 2 diabetes has been reported to be associated with mitochondrial oxidative stress and a depleted state of mitochondrial GSH in rat heart, brain, and kidney [33]. 

## 4. Redox Compartmentation and Communication

Redox regulatory system controls normal cellular functions since changes in oxidation/reduction state of redox couples affect protein conformation, function, interactions, trafficking, and degradation. Moreover, cell compartments have different redox characteristics and, within each compartment, thiol/disulphide control systems are not in thermodynamic equilibrium. Cellular redox biology requires both compartmentation and communication of redox systems so that the thermodynamic disequilibrium of the major redox control nodes or switches (functioning either as sensor or rheostat) allows rapid and sensitive responses to perturbations in redox environments [6]. Cysteine and cystine constitute the major low-molecular-weight thiol/disulphide couple in *human plasma*. The mean plasma Cys/CySS redox value is about −80 mV. This implies that the Cys/CySS couple is not in equilibrium with the plasma GSH/GSSG pool whose redox state is about –140 mV. Plasma also contains intact thioredoxin-1 and its truncated form, both secreted by cells to the extracellular compartment and exhibiting cytokine-like and chemokine-like activities [6,34]. In this regard, it is worth noting that the reduced state of critical cysteines affects many mammalian transactivators. Thioredoxins are involved (by direct and/or indirect pathways) in the regulation of transcription factors, such as AP-1, NFkB, p53, and Sp1, which in turn can regulate expression from the thioredoxin gene promoter [35].

The redox state of *cytoplasm*, measured using cytosolic GSH/GSSG and thioredoxin-1, can be modulated by physiologic stimulation at the plasma membrane. Controlled changes in redox couples potential serve as components for signal transduction, similarly to the phosphorylation cascade. The steady-state redox potential (E_h_) of GSH/GSSG ranges between −193 mV in erythrocytes (which have no intracellular organelles) and −200 mV in cells with nuclei and mitochondria. The E_h_ value of cellular thioredoxin-1 is −280 mV [6]. 

The principal thiol antioxidant systems in *mitochondria* are GSH and thioredoxin-2 system, which depend on the reducing power of NADPH/NADP^+^ [6]. GSH is synthesised in the cytoplasm and then transported into mitochondria by dicarboxylate carrier and 2-oxoglutarate carrier. The E_h_ value of mitochondrial GSH/GSSG has been calculated about -280 mV. The glutathione redox status determines the rate of mitochondrial reactive oxygen species (ROS) production and changes in the absolute concentrations of GSH and GSSG as well as the GSH/GSSG ratio may trigger the reversible opening of an inner membrane anion channel and irreversible permeability transition pore activation [36].

A low rate generation of ROS is normally associated with mitochondrial electron transport activity: ROS can serve as messengers but also damage macromolecules. In order to protect the genome from chemical insults, *nuclei* also possess two antioxidant systems: GSH and thioredoxin-1. Both GSH and thioredoxin-1 could regulate the activity of several transcription factors [37]. A principal regulator of antioxidant response elements (AREs) in gene promoters is the transcriptor factor NF-E2-related factor (NRF) 2. Under normal conditions, NRF2 is a short-lived protein that is readily ubiquitylated in the presence of Kelch-like ECH-associated protein 1 (KEAP1). Upon redox and electrophile stress, KEAP1 is inactivated, thus allowing NRF2 to evade ubiquitylation and accumulate in the nucleus, where it is recruited to gene promoters and transactivates ARE-driven genes [38].

## 5. NO/S-Nitrosothiols Signalling and Vascular Homeostasis

The role of NO as signalling molecule is well established and signalling may occur either by activating guanylate cyclase or, according to current undestanding, by a guanylate cyclase-independent pathway involving S-nitrosylation of cysteine residues in target proteins and formation of *S*-nitrosothiols (SNO). This post-translational modification leads to changes in protein activity, protein-protein interactions, or subcellular localisation of proteins and has been involved in signal transduction [39]. NO synthase (NOS) activity leads directly to SNO formation; NO may react with thiyl radicals or with thiols to form SNO or SNO radicals, respectively. Enzymes other than NOS may catalyze S-nitrosylation, as in the case of coupling of NO and glutathione in the presence of ceruloplasmin [40]. Another example is haemoglobin that plays the enzymatic role in both autonitrosylation of a conserved cysteine as well as S-nitrosylation of the erythrocyte anion exchange protein 1 and low-molecular-weight thiols [40]. A catabolic pathway of SNO, which has been established in knockout mice and plants, is mediated by S-nitrosoglutathione reductase that breaks down cytosolic S-nitrosoglutathione to oxidized GSH and ammonia. *S*-nitrosoglutathione reductase, in turn, modulates the levels of some *S*-nitrosylated proteins by transnitrosylation reactions [40].

NO is an ubiquitous molecule produced by the action of different nitric oxide synthase isoforms on the amino acid arginine. NO binding to the ferrous haeme of the soluble guanylate cyclase in vascular smooth muscle cells and platelets, and consequent enzyme activation, induces vasodilation and inhibition of platelet aggregation. Oxygenated haemoglobin also reacts with NO to form methaemoglobin and nitrate (NO_3_^−^). This inactivating reaction was considered incompatible with the other known capacity of erythrocytes to induce hypoxic vasodilation; several solutions to the paradox have been suggested and extensively debated [41].

Impaired NO availability, due to endothelial dysfunction, is claimed central in cardiovascular disease associated with diabetes mellitus, hypertension, smoking, and metabolic syndrome [42,43]. Advanced glycosylation end product (AGE) formation and their interaction with the receptors for AGE (RAGE) alter vascular function through multiple mechanisms, including oxidative stress and impaired NO availability and bioactivity [44]. Uncoupling of the endothelial NOS in blood vessels of diabetic patients leads to excessive superoxide anion production and diminished NO availability presumably due to reduction of the essential endothelial NOS cofactor tetrahydrobiopterin and involvement of the protein kinase C in exaggerated superoxide production. Moreover, the exposure of endothelial NOS to oxidants including peroxynitrite releases zinc from the zinc-thiolate cluster of eNOS resulting in uncoupling of the enzyme [45]. Disruptions in NO transport and delivery mechanisms in both the erythrocytes and plasma may be present in diabetic subjects [46,47]. 

In studies performed in the mouse and rat, the ventilatory response to hypoxia is regulated in the nucleus tractus solitarius by SNO, specifically S-nitrosoglutathione, exported from deoxygenated erythrocytes [48]. Since aberrant expression of the transcription factor hypoxia inducible factor-1 (HIF-1) could lead to the development of pulmonary artery hypertension and SNO formation has been found to be associated with haemoglobin deoxygenation, it has been hypothesised that signalling through SNO could alter HIF-1 expression and provide an alternative mechanism for the development of this disease. In a randomised double-blind trial, oral treatment with *N*-acetylcysteine (NAC) enhanced the hypoxic ventilatory response that, in turn, correlated with plasma thiol level and plasma thiol-disulphide redox state [49]. Previously unappreciated vascular toxicity of NAC and SNO emerged in mice where systemic administration of NAC, and its conversion product *S*-nitroso-*N*-acetylcysteine, caused hypoxia-mimetic arterial hypertension [50]. 

## 6. Vascular Effects of Sulphydryl-Donor Molecules

Since oxidative stress is thought to contribute to diabetic complications and particularly vascular dysfunction, a growing interest has been given to novel antioxidant therapeutic strategies. Agents that quench ROS, such as vitamin E, C, and alpha lipoic acid appeared promising in animal models and initial human studies, but subsequent larger trials have failed to demonstrate improvement in cardiovascular outcomes. Other drugs, such as statins, angiotensin-converting enzyme inhibitors, angiotensin-receptor blockers, and thiazolinediones, which are used for varied clinical purposes, appeared more effective in improving cardiovascular outcomes probably due to their success in reducing the production of ROS at an earlier part of the cascade [51]. Here we will focus on NAC that has been suggested as a promising treatment in this field. 

NAC has been in clinical use primarily as a mucolytic and chemopreventive agent in chronic obstructive pulmonary diseases and clinical benefits have been usually assumed to result from its ability to deliver cysteine to the portal circulation and thus to restore GSH concentrations. Secondarily, the effects on endothelial function have been investigated with contrasting results. Supplementation with NAC (600 mg/day) lowered homocysteine levels and improved endothelium-dependent dilation in patients with coronary artery disease [52]. On the contrary, oral supplementation with NAC (500 mg/day) neither lowered plasma homocysteine concentrations nor improved flow-mediated dilation of the brachial artery in stable cardiac transplant recipients [53]. Recent observations documented that oral supplementation of NAC (1200 mg/day) plus arginine (1200 mg/day) for six months in patients with type 2 diabetes and hypertension reduced mean systolic and diastolic blood pressure, total cholesterol, LDL cholesterol, oxidised LDLs, high-sensitive C-reactive protein, intercellular and vascular-cell adhesion molecules, nytrotyrosine, fibrinogen, plasminogen activator inhibitor-1, and intima-media thickness. The combined administration increased plasma levels of HDL-cholesterol and nitrites/nitrates. Thus, the intervention seemed able to improve NO bioavailability via reduction of the oxidative stress and increase of NO production [54].

Inconsistencies between experimental and epidemiology studies can be partially explained by a Phase I pharmacokinetic and pharmacodynamic study of NAC [55]. The dose escalation part of the study (dose range 400 to 6400 mg/m^2^/day) indicated that a dose of 800 mg/m^2^/day was at the upper limit of tolerability but produced measurable pharmacodynamic effects; thus, it was selected for daily administration in the constant dose part of the study (6 months). At a dose of 800 mg/m^2^/day, there was an extensive intersubject variation in plasma NAC levels possibly contributed by differences in the rate of NAC deacylation in the intestinal mucosa as well as in the degradation process. At the first visit the C_max_ of NAC in plasma varied from 10.4 to 127.45 μmol/L, and after the final dosing it was 9.8-47.8 μmol/L. In the 3-week protocol of Palmer et al [48] mice, which developed hypoxia-mimetic pulmonary artetial hypertension, had achieved serum NAC levels of 16.2 ± 4.3 μmol/L. 

An in vitro assessment of the effects of different concentrations of NAC (0-5 mmol/L) on the action potentials of sciatic nerve fibers of rats showed a dose-dependent acute inhibition. Since ROS at very low concentrations have a role in cell function, complete deprivation of ROS was supposed to interfere with the proper functioning of nerve fibers. Paradoxically, NAC at 1 mmol/L caused 100% neuroprotection against cadmium-induced neurotoxicity [56]. In patients with type 2 diabetes mellitus, the administration of NAC was able to reduce both the post-prandial oxidative status and endothelial activation [57].

The dual toxic-protective property of NAC in experimental settings proposes again the general problem of designing antioxidants for therapeutic use. Indeed, the basic principles of free radical chemistry suggest that (1) chain-breaking antioxidants can exert pro-oxidant properties and accelerate oxidative damage under certain circumstances; and (2) a complex interaction exists between extra- and intra-cellular redox control. Although NAC appears promising in some clinical settings, long-term randomised clinical trials are still required to evaluate both the efficacy and the safety of chronic NAC administration in humans taking into account some unexpected adverse effects that have been observed in vitro and in animals.

## 7. Perspectives in Diabetes Research

Cardiovascular disease is the major cause of morbidity and mortality for individuals with diabetes, yet reducing cardiovascular risk factors can effectively prevent or slow cardiovascular disease in diabetic people. 

Signal sensing and transduction operate by post-translational modification of proteins. Modulation of thiol-based redox switches in enzymes, receptors, transport proteins, and transcription factors is recognized as an important mechanism of signal transduction, whose dysregulation as a consequence of oxidative stress is associated with cardiovascular disease in diabetes mellitus. Unfortunately, the molecular mechanisms underlying thiol-based redox control remain poorly defined [58]. Particular attention has been paid to the oxidation of cysteine ligands in proteins harbouring Zn^2+^ sites and consequent conformational/functional changes an example of which is uncoupling NO synthesis [59]. 

Insulin exerts both metabolic and haemodynamic effects, the latter ones being primarily mediated by increased NO availability [43]. Insulin resistance is associated with impaired endothelial-mediated vasodilation due both to insulin inability to stimulate NO synthesis and enhanced NO consumption. In turn, endothelial dysfunction is both a cause and a consequence of the metabolic abnormalities typical of insulin resistant states. Alterations in vascular homeostasis have an impact on the expression of vascular and intercellular adhesion molecules, the regulation of procoagulant and anticoagulant properties of the vessel wall, and the maintenance of oxidant/antioxidant balance.

Oxidant stress further amplifies the inflammatory process by upregulating adhesion molecules, inflammatory cytokines and chemokines. Therapies aimed simultaneously at improving (1) carbohydrate and lipid metabolism; (2) insulin resistance; (3) vascular function; (4) blood pressure; and (5) procoagulant and inflammatory responses can reduce cardiovascular morbidity and mortality in high-risk adults. However, the balance between the benefits and harms of every long-term medical therapy needs to be accurately assessed. 
